# High prevalence of antibodies against polyomavirus WU, polyomavirus KI, and human bocavirus in German blood donors

**DOI:** 10.1186/1471-2334-10-215

**Published:** 2010-07-20

**Authors:** Florian Neske, Christiane Prifert, Barbara Scheiner, Moritz Ewald, Jörg Schubert, Andreas Opitz, Benedikt Weissbrich

**Affiliations:** 1 Institute of Virology and Immunobiology, University of Würzburg, Versbacher Str. 7, 97078 Würzburg, Germany; 2 Institute of Transfusion Medicine and Hemotherapy, University Clinic Würzburg, Josef-Schneider-Str. 2, 97080 Würzburg, Germany

## Abstract

**Background:**

DNA of the polyomaviruses WU (WUPyV) and KI (KIPyV) and of human bocavirus (HBoV) has been detected with varying frequency in respiratory tract samples of children. However, only little is known about the humoral immune response against these viruses. Our aim was to establish virus-specific serological assays and to determine the prevalence of immunoglobulin G (IgG) against these three viruses in the general population.

**Methods:**

The capsid proteins VP1 of WUPyV and KIPyV and VP2 of HBoV were cloned into baculovirus vectors and expressed in Sf9 insect cells. IgG antibodies against WUPyV VP1, KIPyV VP1, and HBoV VP2 were determined by immunofluorescence assays in 100 plasma samples of blood donors.

**Results:**

The median age of the blood donors was 31 years (range 20 - 66 yrs), 52% were male. 89% of the samples were positive for WUPyV IgG (median age 31 yrs, 49.4% male), 67% were positive for KIPyV IgG (median age 32 yrs, 46.3% male), and 76% were positive for HBoV IgG (median age 32 yrs, 51.3% male). For WUPyV and HBoV, there were no significant differences of the seropositivity rates with respect to age groups or gender. For KIPyV, the seropositivity rate increased significantly from 59% in the age group 20 - 29 years to 100% in the age group > 50 years.

**Conclusions:**

High prevalences of antibodies against WUPyV, KIPyV, and HBoV were found in plasma samples of healthy adults. The results indicate that primary infection with these viruses occurs during childhood or youth. For KIPyV, the seropositivity appears to increase further during adulthood.

## Background

Infections of the respiratory tract are a major cause of human morbidity. They are most often caused by respiratory viruses, which include the well-known pathogens respiratory syncytial virus, influenza viruses A and B, adenoviruses, parainfluenza viruses, rhinoviruses, and coronaviruses. In recent years, a number of unknown viruses have been identified in the respiratory tract by molecular methods, including the human metapneumovirus, several corona viruses, the human bocavirus (HBoV), and most recently the polyomaviruses WU (WUPyV) and KI (KIPyV) [1-4].

Initial studies on WUPyV and KIPyV have looked at genome detection rates using polymerase chain reaction (PCR) methods. The genoprevalences for WUPyV and KIPyV in respiratory samples from children with acute respiratory tract diseases were found to range from 1.1 to 7.0% [4-10] and 0.9 to 2.7% [3,6-8,11], respectively. However, WUPyV and KIPyV DNA were found at similar frequencies in control groups without respiratory tract disease [5,8,11]. Therefore, the clinical relevance of WUPyV and KIPyV infections is currently unclear. In contrast to PCR assays, serological assays for antibody detection against HBoV are more complex to establish. However, determining immunoglobulin (Ig) M and IgG antibodies in appropriate serum or plasma samples allows to define the point in time of primary infection as well as exposure rates. One study describing the seroepidemiology of polyomaviruses including WUPyV and KIPyV in adults has recently been published [12].

HBoV is a virus of worldwide distribution. Its DNA has been found in 1.5% to 19% of respiratory secretions from children with acute respiratory tract diseases using PCR [13]. Elucidation of the clinical relevance of HBoV has been difficult because of a high co-infection rate of HBoV DNA positive samples with other respiratory viruses. Based on the combination of a high HBoV load in nasopharyngeal samples (> 10^4 ^copies/ml) and concomitant HBoV DNA detection in sera, a model has been proposed in which HBoV is associated with acute respiratory tract diseases but persists in the respiratory tract for a longer period of time than other respiratory viruses after primary infection [14]. Recent serological studies have demonstrated evidence of primary HBoV infection in children with acute respiratory tract diseases, strongly indicating that HBoV is indeed a respiratory pathogen [15,16]. In seroprevalence studies from Japan, the United States, China, and Germany the proportions of HBoV IgG-positive samples increased with age during infancy until reaching levels of > 80% at the age of > 4 years [15,17-19].

In order to expand the epidemiological knowledge about HBoV, WUPyV, and KIPyV, we expressed capsid proteins in the baculovirus system and established an immunofluorescence assay (IFA) for the detection of IgG antibodies against these three viruses. We used this system to determine the prevalence of antibodies against HBoV, WUPyV, and KIPyV in healthy adults.

## Methods

### Blood donor samples

The specimens tested for WUPyV, KIPyV, and HBoV serology consisted of 100 consecutive plasma samples of healthy blood donors received in 2006 from the Institute of Transfusion Medicine and Hemotherapy at the University Clinic of Würzburg. The median age of the blood donors was 31.5 years (range 20.4 - 66.3 years) and 52% were male. The samples were screened routinely for infectious diseases transmitted by blood (human immunodeficiency virus, hepatitis B virus, hepatitis C virus, syphilis). Remaining material was stored at -20°C until use. The study was carried out in compliance with the Helsinki Declaration. Informed consent of the blood donors was obtained. According to the ethics committee of the medical faculty at the University of Würzburg, formal approval of the study was not necessary because the samples were tested in an anonymised fashion.

### Protein expression

The VP2 gene of HBoV was amplified from a nasopharyngeal aspirate using the primers BoV3446s and BoV5113a and Pfu Polymerase (Fermentas, St. Leon Rot, Germany) resulting in a 1667 bp PCR product (Table 1). Similarly, the VP1 genes of WUPyV and KIPyV were amplified from nasopharyngeal aspirates with the primer pairs WU1673s/WU2782a and KI1501s/KI2727a resulting in amplicons of 1109 bp and 1226 bp, respectively (Table 1). Sequences were submitted to GenBank with the accession numbers FJ560720 (HBoV), EU711057 (WUPyV), and FJ647575 (KIPyV). For production of the recombinant baculoviruses BacWUVP1, BacKIVP1, and BacBOVP2 the LR recombination system (Invitrogen, Karlsruhe, Germany) was used according to the recommendations of the manufacturer. Briefly, the amplicons were inserted by TOPO cloning into the pENTR vector (Invitrogen) and subsequently transformed in chemically competent *E.coli *TOP10 cells (Invitrogen). The resulting plasmids pBOVP2, pWUVP1, and pKIVP1 were extracted from overnight culture with the QIAprepSpin Miniprep Kit (Qiagen, Hilden, Germany) and sequenced by standard techniques to confirm orientation and sequence identity of the plasmid insert. The plasmid inserts were then transferred into baculoviral DNA by homologous recombination based on Gateway technology and the BaculoDirect system (Invitrogen). The resulting recombinant baculovirus DNA was directly transfected to adherent Sf9 cells in one well of a six-well-plate using Cellfectin reagent (Invitrogen). The cells were kept in TC-100 growth medium (Lonza, Basel, Switzerland) supplemented with 10% fetal calf serum, 100 U/ml penicillin, 100 μg/ml streptomycin, 0.25 μg/ml amphotericin B, and 100 μM ganciclovir as selective reagent. After five days, the cell culture supernatant was collected and used to infect fresh Sf9 cells. This procedure was repeated twice, resulting in the supernatant of passage 4 (P4), which was finally collected and stored at 4°C for further use.

**Table 1:** Primers used for the Amplification of WUPyV VP1, KIPyV VP1, and HBoV VP2

Primer	**Sequence (5' - 3')**^**a**^	Gene	**Position**^**b**^	Virus
WU1673s-TOPO	CACCGCCTGCACAGCAAAGC	VP1	1673-1688	WUPyV
WU2782a	ACATTATCCTTGTGTGTTTAGTATTGGGCC	VP1	2782-2753	WUPyV
KI1501s-TOPO	CACCAGCTGCACCCCGTGT	VP1	1501-1519	KIPyV
KI2727a	CCTTACTGAGTTTGCCACTATGCA	VP1	2727-2704	KIPyV
BoV3446s-TOPO	CACCTCTGACACTGACATTCAAGAC	VP2	3446-3466	HBoV
BoV5113a	AGGAGGAACTTGTAAGCAGAAGC	VP2	5113-5091	HBoV

^a ^Nucleotides added for directional cloning are underlined.

^b ^Positions according to GenBank accession number EU711057 (WUPyV), NC_009238 (KIPyV), and DQ000496 (HBoV).

Protein expression was confirmed by sodium dodecyl sulfate polyacrylamide gel electrophoresis (SDS-PAGE) and immunoblotting. To this end, Sf9 cells at approximately 80% confluence were inoculated with recombinant virus of P4. The cells were observed daily until at least 80% of the cells displayed a cytopathic effect, which typically happened after seven days. Subsequently, the cell culture supernatant was collected and centrifuged with 3400 g at 4°C for 5 min to remove cells and large debris. The samples were separated by 10% SDS-PAGE and blotted on a nitrocellulose membrane by standard procedures. After blocking, immunodetection was performed with HRP-conjugated mouse-anti-V5-antibody (Invitrogen) at a concentration of 188 ng/ml. Bands were visualised by SuperSignal West Pico chemiluminescence substrate (Pierce, Rockford, USA). Uninfected Sf9 cells were used as negative control.

### Immunofluorescence assay

Sf9 cells were inoculated with recombinant baculoviruses and observed daily until approximately 80% of the cells displayed a cytopathic effect. The cells were harvested and centrifuged for 5 min at 1000 g. The supernatant was discarded and the cell pellet was washed three times in phosphate buffered saline (PBS). The Sf9 cells were spotted on glass slides, air dried, fixed with cold acetone and stored at -20°C until use. Slides with uninfected Sf9 cells were prepared in the same manner and were used for control staining to detect anticellular antibodies. One spot each with Sf9 cells expressing WUPyV VP1, KIPyV VP1, or HBoV VP2 and one spot with uninfected Sf9 cells were incubated with a 1:10 dilution of each plasma sample for 2 h at 37°C. Subsequently, the slides were washed twice for 5 min with PBS containing 0.1% Tween 20 followed by a short rinse in PBS without Tween 20. Next, the slides were incubated with a 1:40 dilution of fluoresceine-conjugated goat-anti-human-IgG (Invitrogen) and a 1:80 dilution of evans blue (Mast Diagnostik, Reinfeld, Germany) for 1 h at 37°C. After another washing step as described above, coverslips were mounted for immunofluorescence microscopy. The slides were independently read by two experienced investigators. Plasma samples, which exhibited a vesicular fluorescence adjacent to the membrane of Sf9 cells, were recorded as antibody positive, if no staining of the control cells was detected.

### Absorption test

For the absorption test, three plasma samples were selected that were positive for antibodies against WUPyV, KIPyV, and HBoV. Sf9 cells infected with each of the recombinant baculoviruses (BacWUVP1, BacKIVP1, BacBoVP2) were harvested from small flasks and resuspended in 500 μl PBS. The suspensions were sonicated for 10 s on ice with a sonicator (Branson Sonifier 250). Each of the three plasma samples was diluted 1:5 in each of the cell lysates. After a 6 h of incubation on ice, the samples were centrifuged at 1400 g for 5 min and the supernatants were diluted in PBS to a final sample concentration of 1:10. Twofold dilution series ranging from 1:10 to 1:1280 of the absorbed and unabsorbed plasma samples were tested in parallel by IFA as described above.

### Statistical analysis

Statistical analysis was carried out using GraphPad Prism version 3.0c for Mac (GraphPad Software, San Diego, USA) and SPSS version 16 for windows (SPSS, Chicago, USA).

## Results

**Figure 1:**
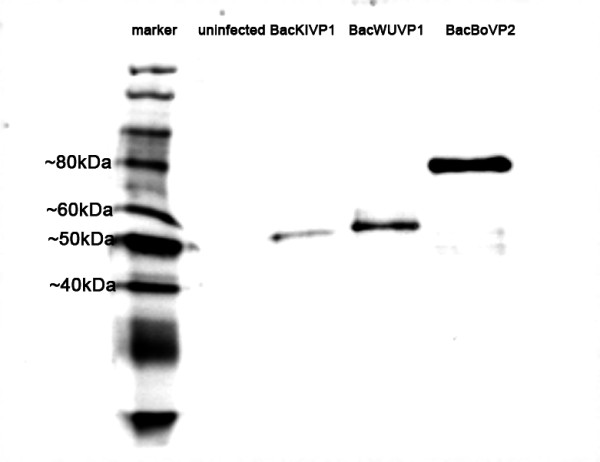
**Confirmation of expression of recombinant proteins**. Sf9 cells were infected with recombinant baculoviruses BacWUVP1, BacKIVP1, and BacBoVP2. Uninfected Sf9 cells served as negative control. After SDS-PAGE and immunoblot, proteins were visualised by staining with anti-V5 antibody. The expected protein sizes are ~46 kDa (WUPyV and KIPyV VP1) and ~66 kDa (HBoV VP2).

Capsid proteins of WUPyV, KIPyV, and HBoV were expressed by infection of Sf9 cells with the recombinant baculoviruses BacWUVP1, BacKIVP1, and BacBOVP2. As antibodies against these proteins are not yet available, the correct size of the expressed proteins was confirmed by SDS-PAGE and immunoblotting using an anti-V5 antibody. The V5-epitope is located upstream of the inserted gene in the baculovirus DNA. A band of the expected size (~46 kDa for WUPyV and KIPyV VP1; ~66 kDa for HBoV VP2) was observed for all recombinant baculoviruses, whereas no band was observed in the uninfected control (Figure 1).

**Figure 2:**
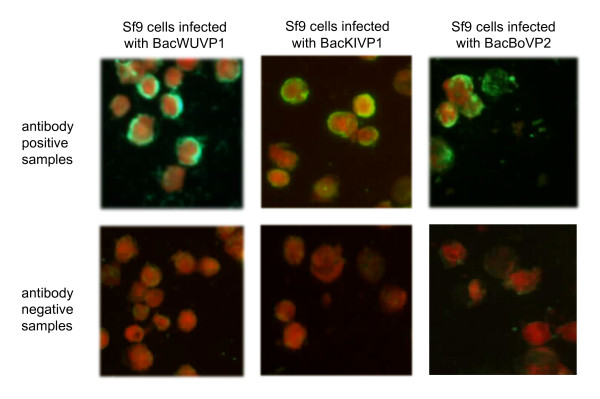
**IFA for the detection of IgG antibodies against WUPyV VP1, KIPyV VP1, and HBoV VP2**. Sf9 cells infected with recombinant baculoviruses were incubated with plasma samples of healthy blood donors. Bound antibodies were visualised with FITC-labelled anti-human IgG. Representative examples of positive and negative plasma samples are shown for each of the three antigens.

To determine the frequency of past exposure with WUPyV, KIPyV, and HBoV in healthy adults, 100 blood donor plasma samples were tested for the presence of IgG against WUPyV VP1, KIPyV VP1, and HBoV VP2 using an IFA based on Sf9 cells infected with the recombinant baculoviruses (Figure 2). All samples were also tested by IFA using uninfected Sf9 cells. No positive reaction with cellular antigens was observed in any of the samples. In order to further confirm the specificity of positive IFA results, three plasma samples were studied in an absorption assay. Antibody titers were determined before and after absorption with lysates of Sf9 cells infected with recombinant baculoviruses. A significant titer decrease was only observed after absorption with the matching Sf9 cell lysate (Table 2). There was no indication of antibody cross-reaction between Sf9 cells infected with BacWUVP1, BacKIVP1, and BacBOVP2.

**Table 2:** Antibody titers before and after absorption with lysates of virus capsid protein expressing Sf9 cells

		**Antibody titers after absorption with lysates of Sf9 cells expressing**^**a**^		
		
Plasma sample and antibody	Titers prior to absorption	WUPyV VP1	KIPyV VP1	HBoV VP2
**Patient 1**				
Anti-WUPyV	1:80	**1:20**	1:80	1:40
Anti-KIPyV	1:80	1:80	**1:20**	1:80
Anti-HBoV	1:160	1:160	1:160	**1:10**
**Patient 2**				
Anti-WUPyV	1:40	**1:10**	1:20	1:20
Anti-KIPyV	1:320	1:320	**1:40**	1:320
Anti-HBoV	1:320	1:640	1:640	**1:40**
**Patient 3**				
Anti-WUPyV	1:160	**1:40**	1:160	1:80
Anti-KIPyV	1:160	1:80	**1:20**	1:160
Anti-HBoV	1:320	1:320	1:320	**1:10**

^a ^Antibody titers obtained with homologous Sf9 cell lysates are shown in bold.

**Figure 3:**
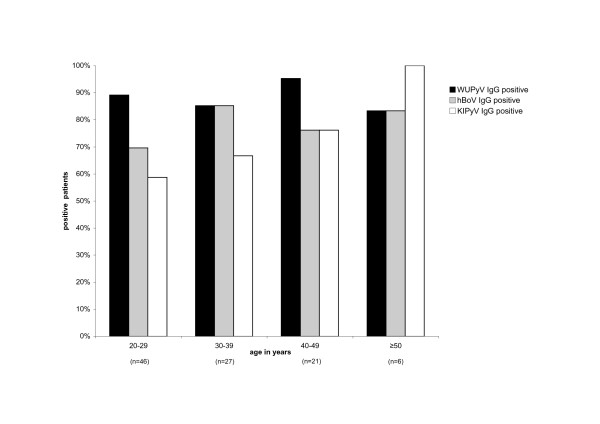
**Age distribution of virus-specific IgG antibodies**. The seroprevalence for WUPyV and HBoV did not differ significantly between age groups (p = 0.855 and p = 0.175, respectively). For KIPyV, a significant rise of seroprevalence rates was observed with increasing age (p = 0.026).

Of all plasma samples, 89% were positive for anti-WUPyV VP1 IgG, 67% were positive for anti-KIPyV VP1 IgG, and 76% were positive for anti-HBoV VP2 IgG. Neither the median age nor the gender distribution of the antibody positive samples were significantly different from the total population for any of the three viruses tested (Mann-Whitney test and Fisher's exact test, respectively; Table 3). For WUPyV and HBoV, there were no significant differences of the seropositivity rates with respect to age groups. For KIPyV, the seropositivity rate increased significantly from 58,7% in the age group 20 - 30 years to100% in the age group > 50 years (p = 0.026; chi-square test for trend; Figure 3).

**Table 3:** IgG seroprevalence for HBoV VP2, WUPyV VP1, and KIPyV VP1 in 100 healthy blood donors

group	n	Median age in years	Male
All samples	100	31.5	52%
WUPyV IgG positive	89	31.2	49%
KIPyV IgG positive	67	32.1	46%
HBoV IgG positive	76	31.8	51%

Table 4 shows the number of samples grouped by individual results for WUPyV, KIPyV, and HBoV IgG. One percent of the samples were negative for all three viruses and 50% were positive for all three viruses. No obvious cross-reaction between the three antibody specificities was apparent.

**Table 4:** Results of WUPyV, KIPyV, and HBoV IgG IFA of individual samples

	Result of HBoV IgG IFA (n)		
Results of WUPyV and KIPyV IgG IFA	Negative	Positive	Total (n)
WUPyV and KIPyV negative	1	3	4
WUPyV negative, KIPyV positive	1	6	7
WUPyV positive, KIPyV negative	12	17	29
WUPyV and KIPyV positive	10	50	60
Total	24	76	100

## Discussion

Using IFA based on insect cells expressing capsid proteins of WUPyV, KIPyV, and HBoV, we studied the seroprevalence of these viruses in plasma samples of healthy adults. We found high rates of positive IgG antibodies against all three viruses: 89% for WUPyV, 67% for KIPyV, and 76% for HBoV. Our data on seroprevalence for WUPyV and KIPyV are somewhat higher than reported in a recent study from the United States, which found seroprevalences of 54.1% to 55.3% for KIPyV and 67.4% to 70.9% for WUPyV in the age groups from 21 to 70 years of North American blood donors [12]. In contrast to our findings, a rising prevalence for KIPyV IgG in adults was not observed. Further studies are necessary in order to determine whether there may be regional differences in the KIPyV epidemiology. Taken together, the data of Kean et al. and of our study indicate that WUPyV and KIPyV have a widespread distribution similar to the well-known polyomaviruses BKV and JCV. Also in agreement with BKV and JCV epidemiology, primary exposure seems to occur mainly in childhood and youth. Primary BKV infection has been reported to occur mainly in the first decade of life leading to a seroprevalence of 65% to 90% at the age of 10 (reviewed in [20]). The JCV seroprevalence in adults has been found between 44% and 97%. Whereas some studies reported a time frame for primary JCV infection similar to BKV infections, others showed a continuing rise of the JCV seroprevalence during adulthood (reviewed by [20]).

VP1 is the major capsid protein of polyomaviruses. Several studies on BKV and JCV serology have used this protein successfully in enzyme immunoassays, immunoblots, or IFA [21-24]. Therefore, we decided to use expression of VP1 proteins to establish IFAs for the detection of WUPyV and KIPyV IgG. In the study by Kean et al., WUPyV and KIPyV antibodies were determined by an enzyme immunoassay based on bacterially expressed VP1 capsomeres [12].

Validation of these IFA was limited by the lack of defined serum samples that could be used to determine assay sensitivity and specificity. By testing selected study samples before and after absorption with lysates of Sf9 cells that were infected with different recombinant baculoviruses, we were able to exclude general cross reactivity between WUPyV and KIPyV. Furthermore, we excluded anticellular reactivity of the plasma samples by performing an IFA with uninfected Sf9 cells. Therefore, we are confident that our serological data are reliable. Comparison of different serological methods and antigen preparations as well as sample exchange will be useful to further validate the assays for WUPyV and KIPyV antibody determination.

As to HBoV, the adult seroprevalence of 76% in our study is slightly lower than in previous studies from Japan, the United States, and Germany, which reported HBoV IgG antibody prevalence of 94%, 86%, and 94%, respectively [15,17,19]. The total number of adult samples tested was small in two of these reports (34, 7, and 299, respectively). Whether methodological issues or differences in the study populations account for the lower seroprevalence observed in this study, could be addressed by method comparisons and sample exchange.

We chose the HBoV VP2 gene for recombinant expression on the basis of current knowledge about immunodominant antigens of parvovirus B19, which belongs to the same virus family as HBoV. Previous studies on HBoV serology have used IFA based on VP1-expressing insect cells [15], enzyme immunoassay based on VP2 purified from insect cells [17,18], immunoblot based on VP2 and the unique region of VP1 expressed in *E. coli *[16], and enzyme immunoassay based on VP2 virus-like particles [19]. In the study of Kantola et al., a higher immunoreactivity was demonstrated for HBoV VP2 than for the unique part of HBoV VP1. Overall, the VP2 protein seems to be suitable for use in serological assays. However, a comparison of the VP1 and VP2 protein has not yet been performed.

Limitations of the HBoV IFA validation are similar to the limitations described for the WUPyV and KIPyV IFA. Results of a control IFA using uninfected Sf9 cells and of pre-absorption experiments suggest that the positive HBoV IgG results are specific. Recently, novel bocaviruses related to HBoV have been described in humans [25,26]. If there are cross-reactions between antibodies against the different HBoV types is not known at present and needs to be further investigated.

In this seroepidemiological study, only IgG antibodies were determined. Evidently, the IFAs used in this study may easily be modified for the determination of IgM antibodies. IgG and IgM serology applied on appropriate sample collections will allow to determine the point in time of primary infection. Information on this issue is important in order to establish the clinical relevance of WUPyV, KIPyV, and HBoV.

## Conclusions

Antibodies against HBoV, WUPyV, and KIPyV were found at high rates in sera of healthy German adults. These results suggest that primary infections with these viruses occur mainly during childhood and youth. For KIPyV, the seropositivity appears to increase further during adulthood.

## Competing interests

The authors declare that they have no competing interests.

## Authors' contributions

BW and FN designed and coordinated the study. FN and ME produced the recombinant baculoviruses. FN, CP, BS, ME, and JS established and performed the IFA testing. AO collected the blood donor samples. All authors participated in the data analysis. BW and FN drafted the manuscript. All authors read and approved the final version of the manuscript.

## Pre-publication history

The pre-publication history for this paper can be accessed here:

http://www.biomedcentral.com/1471-2334/10/215/prepub
